# HFA-Net: Explainable Multi-Scale Deep Learning Framework for Illumination-Invariant Plant Disease Diagnosis in Precision Agriculture

**DOI:** 10.3390/s26072067

**Published:** 2026-03-26

**Authors:** Muhammad Hassaan Ashraf, Farhana Jabeen, Muhammad Waqar, Ajung Kim

**Affiliations:** 1 Department of Computer Science, COMSATS University, Islamabad 45550, Pakistan; farhanakhan@comsats.edu.pk; 2 Department of Computer Science, Faculty of Arts and Sciences, Edge Hill University, Ormskirk L39 4QP, UK; waqarm@edgehill.ac.uk; 3 Department of Optical Engineering, Sejong University, Seoul 05006, Republic of Korea

**Keywords:** computer vision in precision agriculture, plant disease classification, deep learning for agriculture, Convolutional Neural Networks (CNN), hybrid CNN, class imbalance in deep learning, attention mechanisms in CNNs, multi-scale residual learning, Test-Time Augmentation (TTA), Explainable Artificial Intelligence (XAI)

## Abstract

Robust plant disease detection in real-world agricultural environments remains challenging due to dynamic environmental conditions. Accurate and reliable disease identification is essential for precision agriculture and effective crop management. Although computer vision and Artificial Intelligence (AI) have shown promising results in controlled settings, their performance often drops under lesion scale variability, inter- and intra-class similarity among diseases, class imbalance, and illumination fluctuations. To overcome these challenges, we propose a Heterogeneous Feature Aggregation Network (HFA-Net) that brings together architectural improvements, illumination-aware preprocessing, and training-level enhancements into a single cohesive framework. To extract richer and more discriminative features from the early layers of the network, HFA-Net introduces a multi-scale, multi-level feature aggregation stem. The Reduction-Expansion (RE) mechanism helps preserve important lesion details while adapting to variations in scale. Considering real agricultural environments, an Illumination-Adaptive Contrast Enhancement (IACE) preprocessing pipeline is designed to address illumination variability in real agricultural environments. Experimental results show that HFA-Net achieves 96.03% accuracy under normal conditions and maintains strong performance under challenging lighting scenarios, achieving 92.95% and 93.07% accuracy in extremely dark and bright environments, respectively. Furthermore, quantitative explainability analysis using perturbation-based metrics demonstrates that the model’s predictions are not only accurate but also faithful to disease-relevant regions. Finally, Grad-CAM-based visual explanations confirm that the model’s predictions are driven by disease-specific regions, enhancing interpretability and practical reliability.

## 1. Introduction

Advancements in AI have significantly advanced precision agriculture by enabling systematic, data-driven approaches to crop monitoring, analysis, and management. The world population keeps growing rapidly, which contributes to the high food demand and eventually intensifies the need for accurate, timely, and scalable crop monitoring solutions. The United Nations (UN) estimates that the world population will reach 10 billion in 2050, resulting in a significant rise in food consumption [1], placing substantial pressure on agricultural systems to significantly increase productivity. Current growth rates remain insufficient, particularly in low-income regions, which are lagging behind global targets in productivity improvements. The 2019 global agriculture productivity report indicates that the agricultural productivity of low-income countries is increasing by an average of only 1% per year. To satisfy this rising demand, global agricultural productivity must grow by 1.75 percent every year [1,2]. Within this context, pests and diseases have a great influence on plant yield and quality, directly impacting food security and economic stability, particularly in developing nations where agriculture is a major sector of the economy [3].

**Figure 1:**
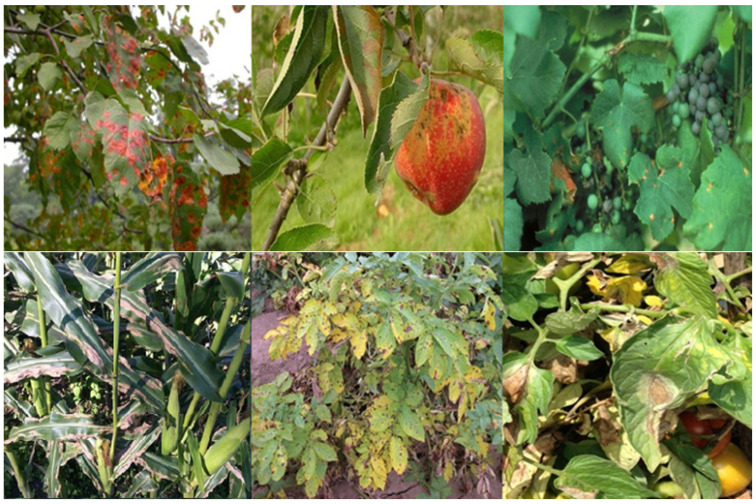
Disease symptoms on plants, leaves and fruits.

Early and reliable disease detection is therefore a cornerstone of precision agriculture, enabling targeted interventions, supporting sustainable farming practices, and reducing unnecessary pesticide use. In agriculture, critical cues for diagnosis are provided by visual symptoms such as discoloration, leaf lesions, and structural abnormalities, which in turn allows the use of automated vision-based disease recognition systems, an essential component of next-generation smart agriculture frameworks. Infected plants are usually characterized by stains or lesions on their leaves, stems, flowers, or fruits as illustrated in Figure 1.

**Figure 2:**
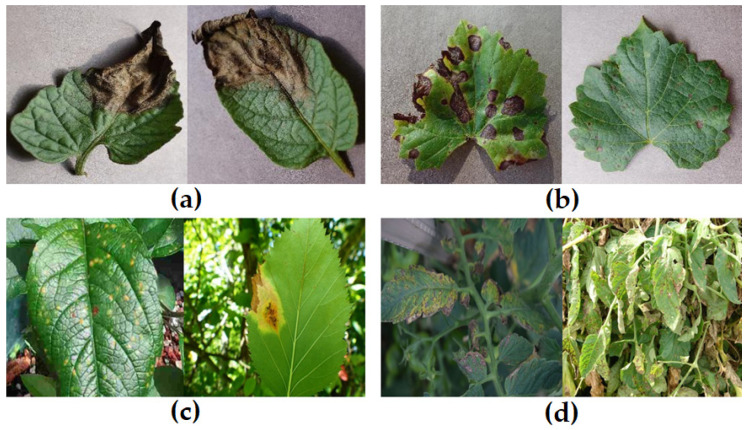
Challenges in plant disease classification. (**a**) Class similarity between tomato and potato early blight, (**b**) lesion-scale variation in grape black rot, (**c**) background clutter in apple rust, (**d**) illumination fluctuation in tomato Septoria leaf spot disease samples.

Researchers have successfully used Machine Learning (ML)-based models that extract handcrafted features and use classifiers like Support Vector Machines (SVMs) or the Naive Bayes for precision agriculture [4]. Nevertheless, these methods tend to be less efficient and slower in disease classification because of the intricacy of feature extraction phases [3]. The remarkable progress in CNNs has shown significant potential in accurately identifying plant diseases [3,4,5,6,7,8]. CNNs automate the feature extraction process, addressing the constraints associated with manual feature extraction [3,9]. Despite significant progress composed with CNNs, these architectures continue to address substantial challenges under real-world conditions, as depicted in Figure 2. Plant disease datasets often contain multiple disease classes, some characterized by similar symptoms and visual appearances, resulting in substantial interclass similarity [5]. Due to the small size of lesions, multiple down-sampling operations in deep feature extraction networks may overlook fine-grained, small-scale patterns. In addition, complex and cluttered backgrounds can lead to false detections due to background noise in leaf images. Furthermore, inconsistent illumination and contrast variations, caused by fluctuating sunlight, shadows, and non-uniform imaging conditions, can severely degrade image quality and obscure critical features, thereby reducing the robustness and generalization capability of existing CNN architectures [8,10].

Traditional CNN architectures, such as VGG16 and AlexNet, rely on fixed-size filters and max-pooling operations, leading to information loss in the early layers and significantly limiting the model’s ability to capture detailed features across different scales [5,11]. More advanced models like DenseNet and ResNet incorporate dense and residual connections to enhance feature flow and mitigate the vanishing gradient problem [3]. However, their convolutional layers mainly rely on fixed kernel sizes (e.g., 3×3), which may limit their ability to effectively capture lesion-scale variations in plant disease images, where symptoms can appear as small scattered spots or large infected regions [5]. Additionally, the use of traditional activation functions, such as Rectified Linear Unit (ReLU), can exacerbate the vanishing gradient issue, leading to dead neurons and restricting the model’s learning capacity [10].

To address the aforementioned challenges, this research proposes an illumination-adaptive and explainable deep learning (DL) framework for robust plant disease classification under real-world conditions. The proposed pipeline mitigates illumination fluctuations by standardizing input images through a Contrast-Limited Adaptive Histogram Equalization and Adaptive Gamma Correction (CLAHE-AGC) enhancement process. These standardized inputs are subsequently used for model training and evaluation, ensuring consistent visual quality across varying lighting conditions.

For robust feature extraction capable of handling lesion-scale variation, inter-class variability, and background clutter, we introduce the HFA-Net, which extracts multi-scale heterogeneous features to capture fine-grained patterns compared to fixed-scale representations, thereby improving diagnostic accuracy across lesions of different sizes. The multi-level feature aggregation mechanism further enhances the network’s representational capacity by capturing complex visual cues and alleviating gradient vanishing or exploding problems [12,13], which strengthens the model’s discriminative ability across disease categories.

To reduce the impact of background clutter, an attention mechanism is integrated to prioritize the most relevant foreground lesion features. During training, a mean augmentation class balancing strategy and focal loss function are employed to counter dataset imbalance, improving generalization to underrepresented classes. Moreover, HFA-Net incorporates the Hard-Swish activation function and Global Average Pooling (GAP) layers [14] to prevent overfitting and maintain stable convergence. Finally, during inference, Test-Time Augmentation (TTA) is applied to test samples under diverse illumination scenarios.

Moreover, in addition to quantitative evaluation (classification accuracy, precision, recall, and F1-score), we integrate an Explainable Artificial Intelligence (XAI) technique known as Gradient-weighted Class Activation Mapping (Grad-CAM) [15] to visualize the most influential regions contributing to the model’s classification decisions. This interpretability component provides insight into the network’s decision-making process, allowing verification that the model focuses on disease-related lesion areas rather than irrelevant background regions, thereby enhancing the framework’s transparency and reliability for practical deployment [16,17]. The primary contribution of the proposed framework lies in its ability to effectively address several critical challenges inherent to the agricultural domain.

- To address illumination variations in real agricultural circumstances, we designed an illumination-adaptive preprocessing pipeline to ensure consistent brightness and contrast across diverse lighting conditions.
- We introduce a novel architecture, HFA-Net, capable of extracting multi-scale heterogeneous features that capture both fine-grained and large-scale disease patterns. The network accommodates varying lesion sizes, enabling accurate diagnosis regardless of patch scale. HFA-Net aggregates multi-level features that effectively capture intricate structural details and mitigate gradient vanishing or exploding problems, thereby enhancing the model’s discriminative capacity across multiple disease categories and improving overall classification accuracy. The proposed multi-scale and multi-level feature extraction enables HFA-Net to maintain high diagnostic accuracy under both high and low illumination conditions, producing illumination-invariant feature representations that improve robustness in field-deployed systems.
- During training, we employ a mean augmentation-based class balancing technique together with an enhanced focal loss function to alleviate dataset imbalance. The integration of the Hard-Swish activation function and GAP layers further prevents overfitting and promotes stable convergence.
- To ensure model transparency and interpretability, Grad-CAM is integrated to visualize the discriminative regions influencing the model’s decisions. This visualization confirms that the network focuses on disease-relevant regions rather than background noise.

Overall, this research paper contributes to precisely identifying plant diseases at an early stage with transparency and improving agricultural productivity.

The remainder of this paper is organized as follows. Section 2 provides a comprehensive review of existing deep learning approaches for plant disease classification, highlighting current limitations under real-world agricultural conditions. Section 3 describes the materials and methods, including dataset construction, the Illumination-Adaptive Contrast Enhancement pipeline, and the architectural design of the proposed HFA-Net. Section 4 presents detailed experimental results, ablation studies, robustness analysis under illumination variability, and comparative evaluations against other CNN models. Finally, Section 5 concludes the paper by summarizing the principal findings and outlining future research directions for robust and explainable vision-based plant disease diagnosis.

## 2. Current State of the Art

This study focuses on DL-based frameworks due to their superior feature representation capabilities compared to traditional ML-based approaches. Early Simple Feed-Forward (SFF) CNN-based methods have been widely adopted for plant disease classification. For instance, Chen et al. [18] employed AlexNet for the classification of tomato leaf diseases. In [19], Latif et al. utilized the Visual Geometry Group (VGG) CNN architecture, specifically VGG19, consisting of nineteen layers, for disease detection in rice plants. The transfer learning technique is applied to fine-tune the model. In [6], Hosny et al. proposed a lightweight CNN architecture for multiclass plant disease classification. Their proposed framework utilizes CNN features and local binary patterns for disease identification. Their lightweight CNN consists of three convolutional, max pooling, and four fully connected layers. These classical architectures overlook the significance of multi-scale and multi-level features, which are crucial for early-stage disease recognition and addressing class variation [17].

To overcome these limitations, recent studies emphasized the importance of multi-scale features. Hussain et al. [20] proposed a maximum correlation feature fusion technique for merging the VGG19 and Inception V3 CNN architectures for cucumber disease diagnosis. VGG19 is a deep feed-forward network known for its ability to extract semantic features through multiple convolutional and max pooling layers. Inception V3 is famous for its capability to extract multi-scale features. Their proposed framework achieved promising results in classifying cucumber leaf diseases. Al-Gaashani et al. [21] modify the MobileNetV2 architecture by utilizing a multi-scale feature extraction mechanism for maize leaf disease classification. To emphasize the importance of multi-scale representations in CNNs, they introduce the PoolFormer block into a truncated version of MobileNetV2. The enhanced network integrates three parallel convolutional branches with varying kernel sizes to effectively capture diverse semantic and spatial features. This multi-scale design yields a slight improvement in classification performance over the baseline MobileNetV2. However, these hybrid multi-scale architectures neglect the extraction of multi-level features vital for smooth gradient flow and address the class variation problem. Moreover, these models utilize the ReLU activation function, which suffers from a problem known as dead neurons, causing them to stop learning and not contribute to the training process [10].

To underscore the importance of multi-level features, Ksibi et al. [22] proposed features concatenation-based olive leaf disease classifier known as MobiResNet. Their proposed framework concatenates the features of MobileNet and ResNet-50 architectures. Their suggested framework extracts multi-level features to classify leaf lesions. Rashid et al. [23] integrate Internet of Things (IoT) sensors with a DL architecture for corn leaf disease classification. Their proposed Multi-Model Fusion Network (MMF-Net) is composed of two major convolutional blocks: the Residual Learning Block (RL-Block), inspired by ResNeXt, and the Perceptual Learning Blocks (PL-Blocks), inspired by AlexNet and VGG16. Alghamdi et al. [3] proposed the PDD-Net architecture for plant disease classification. They combined Dense Connection Block (DCB), to extract multi-level features, and a pyramid pooling module, for obtaining multi-scale features. Taji et al. [24] propose a hybrid DL framework for plant disease classification by leveraging ensemble CNN features and metaheuristic optimization. The proposed framework integrates pre-trained AlexNet and ResNet18 models for deep feature extraction, which are combined with Local Binary Pattern (LBP) texture features to construct a fused ensemble feature vector. A hybrid preprocessing pipeline is introduced, incorporating power-law transformation, CLAHE, LAB color transformation, and K-means clustering to enhance image quality. The enhanced images are then passed through the LBP module, ResNet18, and AlexNet architectures for feature extraction. These hybrid architectures increase model complexity by combining multiple backbones, resulting in higher parameter counts and computational cost. In addition, they generally lack explicit attention mechanisms, which limit the model’s ability to dynamically emphasize disease-relevant regions while suppressing background noise and irrelevant features [25].

To emphasize the significance of the attention mechanism, Dai et al. [7] introduce DFN-PSAN, a multi-level deep information feature fusion network designed to enable reliable plant disease classification. The architecture is based on the YOLOv5 backbone architecture with the addition of a Pyramidal Squeezed Attention (PSA) mechanism to improve its feature extraction capacity. This element is particularly intended to extract and highlight important regions of plant disease images by efficiently combining profound semantic attributes across several convolutional layers. In another study, Dai et al. [8] introduces a new framework, which uses weather-conditioned data augmentation to recreate various environmental conditions, thus improving the diversity of datasets. Their main strategy is PPLC-Net, a CNN architecture that is used to detect plant diseases. PPLC-Net combines dilated convolution, a multi-level attention system, and GAP to enhance feature representation and generalization. One of the most important contributions is the sawtooth dilated convolution with a variable expansion rate that improves the extraction of spatial features. Also, the model uses a Convolutional Block Attention Module (CBAM) to enhance the focus on discriminative features, and GAP is used to avoid overfitting.

Despite notable progress, most of the existing plant disease classification frameworks do not address the combined challenges of illumination variability, lesion scale diversity, class imbalance, and interpretability within a single unified architecture. Conventional CNNs often rely on fixed receptive fields and repeated down-sampling, while hybrid multi-scale and fusion-based methods frequently increase model complexity without sufficiently improving robustness under real-world field conditions. Although attention-based approaches enhance feature selectivity, their explanations are often limited to qualitative visualization without quantitative faithfulness assessment. Moreover, existing studies rarely incorporate systematic TTA-based evaluation under controlled illumination perturbations to measure model robustness across diverse lighting conditions. These limitations motivate the proposed HFA-Net, which integrates illumination-adaptive preprocessing, class-balancing augmentation, heterogeneous multi-scale and multi-level feature extraction, and explainable decision analysis within a compact framework for plant disease diagnosis.

## 3. Materials and Methods

**Figure 3:**
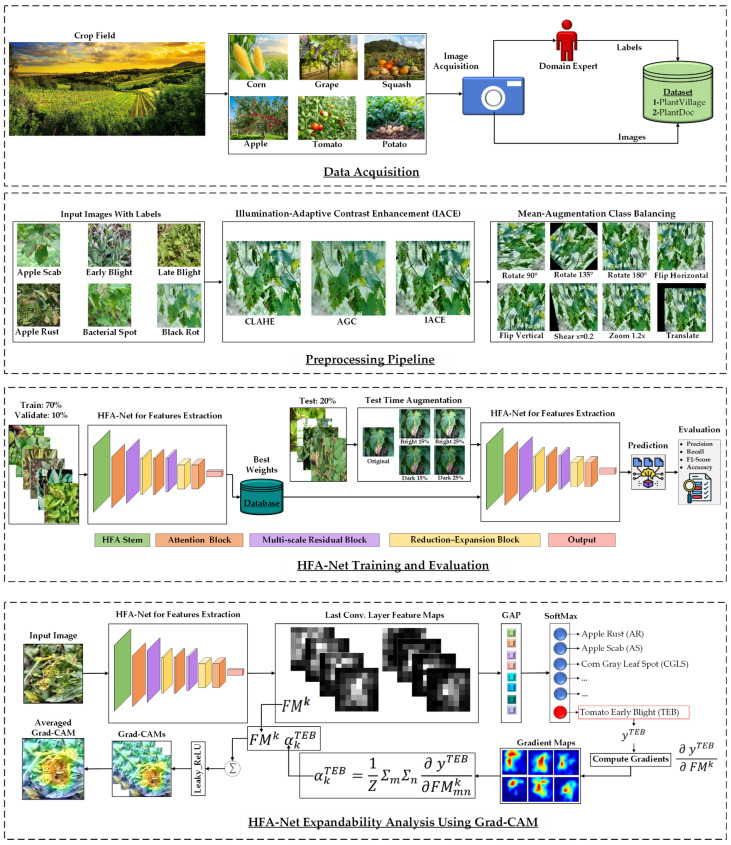
Overview of the proposed plant disease diagnosis framework.

This research presents an illumination-adaptive and explainable framework for Plant Disease Diagnosis (PDD), integrating advanced techniques to enhance accuracy and the model’s interpretability. This section provides an in-depth explanation of each component of the proposed framework, detailing the experimental datasets used for evaluation, and describes the Mean-Augmentation Class Balancing (MACB) strategy employed to address class imbalance. A comprehensive overview of the HFA-Net architecture is provided, focusing on its internal structure for robust and efficient feature extraction. Additionally, this section discusses the specific activation and loss functions used within the HFA-Net model, as well as the integration of Grad-CAM, a tool that enhances the explainability of the framework by visualizing important features in the decision-making process. Figure 3 visually illustrates the complete operational flow of the proposed system.

### 3.1. Benchmark Datasets Acquisition

Publicly available benchmark datasets PlantVillage [26] and PlantDoc [27] were utilized to ensure comprehensive evaluation under both controlled and real-world conditions. The PlantVillage dataset [26] consists of images captured under laboratory-controlled conditions with uniform backgrounds and lighting, which limits its ability to represent real-world variability. In contrast, the PlantDoc dataset [27] contains field-acquired plant disease images that better reflect real agricultural environments. However, it suffers from limited class diversity and insufficient samples per class, making it inadequate for independently training DL models. To overcome these limitations, this study combines common plant disease classes from PlantVillage and PlantDoc to construct the PlantVillageDoc dataset. The PlantVillageDoc dataset comprises 14 disease classes, including Apple Rust (AR), Apple Scab (AS), Corn Gray Leaf Spot (CGLS), Corn Leaf Blight (CLB), Corn Rust Leaf (CRL), Grape Black Rot (GBR), Pepper Bell Leaf Spot (PBLS), Potato Early Blight (PEB), Potato Late Blight (PLB), Squash Powdery Mildew (SPM), Tomato Bacterial Spot (TBS), Tomato Early Blight (TEB), Tomato Late Blight (TLB), and Tomato Septoria Leaf Spot (TSLS).

To prevent potential data leakage and ensure fair evaluation, images from PlantVillage and PlantDoc were first split independently at the class level into 70% training, 10% validation, and 20% testing subsets before merging them to construct the PlantVillageDoc dataset. This strategy ensures that images from the same source dataset do not overlap across training and testing partitions while maintaining balanced class distributions. This integrated dataset incorporates both controlled and real-world samples, providing a more balanced, diverse, and representative benchmark for robust model evaluation.

**Figure 4:**
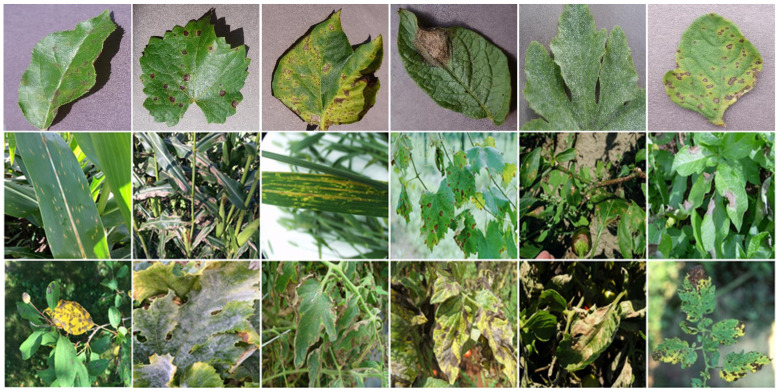
Some samples from PlantVillageDoc dataset.

Some representative samples from the PlantVillageDoc dataset are illustrated in Figure 4.

### 3.2. Mean-Augmentation Class Balancing (MACB)

**Figure 5:**
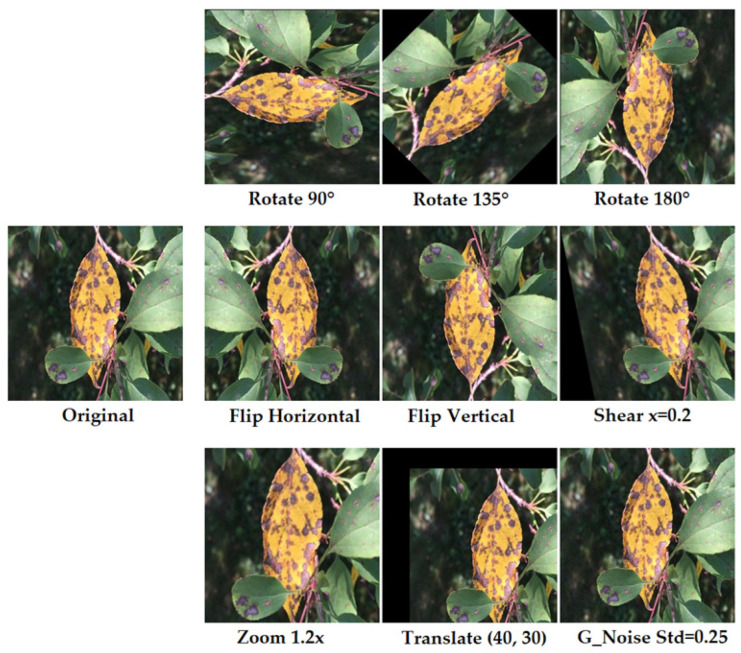
Augmented samples generated from original images using MACB strategy.

The proposed mean augmentation class balancing algorithm addresses dataset imbalance by computing the mean frequency of all classes and using it as a threshold to guide selective augmentation. Classes with sample counts lower than the mean are progressively augmented until their frequency approaches the dataset mean. The algorithm utilizes three sets of augmentation techniques to generate new samples, enhancing the dataset’s diversity and minimizing bias. Some augmented samples are shown in Figure 5. In the first phase, the balancing algorithm applies three different types of rotations, namely, 90°, 135°, and 180°, to augment the data samples. In the second phase, the original images are modified through horizontal and vertical flipping as well as shearing (x = 0.20). Finally, if the number of samples in a particular class remains less than the ‘mean’ of all classes, the third phase is applied, which involves the application of zooming (1.2×), translation (x = 40, y = 30) and random gaussian noise injection with σ = 0.25 to further increase the number of samples. The overall procedure of the proposed MACB algorithm is formally described in Algorithm 1.
**Algorithm 1.** Mean Augmentation Class Balancing (MACB)**Input:** Original dataset, class frequencies, augmentation sets (Set1, Set2, Set3)
**Output:** Augmented (balanced) dataset
  1.  Initialize **AugmentedDataset** as an empty set
  2.  **First augmentation phase:**
     For each class C in the original dataset:      Compute mean class frequency $C_{mean}$      If $C_{mean}$ > frequency of class C:       Apply augmentation techniques from **Set1** to class C       Add augmented images to **AugmentedDataset**

      Else:        Add original images of class C to **AugmentedDataset**

  3.  **Second augmentation phase:**
     For each class C in **AugmentedDataset**:      Compute updated mean class frequency      If mean frequency > frequency of class C:       Apply augmentation techniques from **Set2**       Add new augmented images to **AugmentedDataset**

  4.  **Third augmentation phase:**
     Repeat the above process using augmentation techniques from **Set3**

The details of pre- and post-balancing data samples are described in Table 1.

### 3.3. Illumination-Adaptive Contrast Enhancement (IACE)

**Figure 6:**
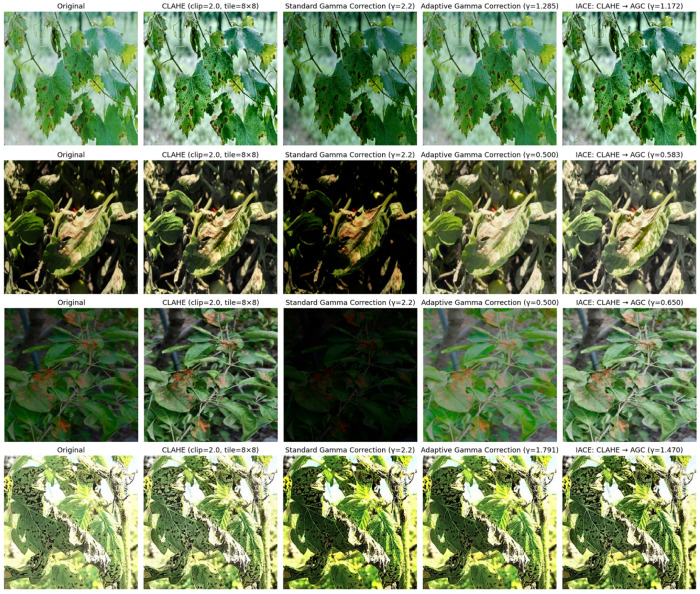
Visual comparison of CLAHE, gamma correction, and the proposed IACE pipeline under varying lighting conditions.

To ensure robustness of plant disease classification under diverse lighting conditions, all input images were preprocessed using an Illumination-Adaptive Contrast Enhancement (IACE) pipeline that integrates CLAHE and AGC. CLAHE was applied in the LAB color space with a clip limit of 2.0 and a tile grid size of 8 × 8, selected after extensive empirical experiments to achieve stable contrast enhancement while preserving lesion details. Increasing these values tended to amplify noise and over-enhance textures, whereas smaller values resulted in insufficient contrast improvement and weaker lesion boundary visibility. Subsequently, AGC dynamically adjusted image luminance by computing the mean brightness and adapting the gamma value within the range [0.5, 2.0], with the target mean brightness set to 0.5, and a small constant ε = 1 × 10^−6^ was added for numerical stability. This preprocessing pipeline minimizes the adverse impact of illumination variability while preserving critical disease-specific visual cues, thereby generating consistent and enhanced inputs for the proposed HFA-Net model. As illustrated in Figure 6, the proposed IACE pipeline enhances contrast and normalizes illumination while preserving disease-relevant features.

### 3.4. Proposed HFA-Net Architecture

**Figure 7:**
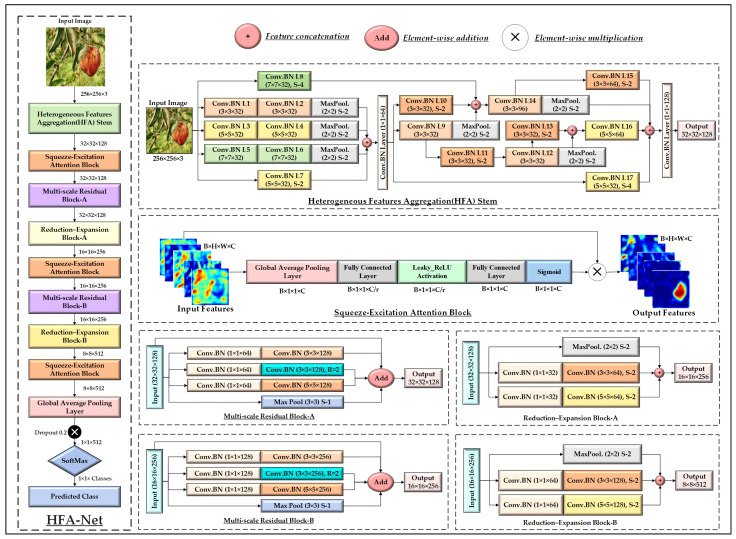
The proposed HFA-Net architecture.

The HFA-Net architecture is designed for processing color images with dimensions of 256 × 256 × 3 and achieves robust classification of plant diseases using heterogeneous feature extraction with attention mechanism, as shown in Figure 7. Unlike conventional CNNs, which are based on an SFF stem, HFA-Net presents a parallel multi-scale and multi-level HFA stem, which effectively captures various spatial patterns at the early layers of the network. The HFA stem uses three parallel convolutional branches with different kernel sizes to capture fine-to-coarse visual patterns. The first branch consists of two consecutive 3 × 3 × 32 Convolution-Batch-Normalization (Conv.BN L1 and Conv.BN L2) operations followed by a 2 × 2 max-pooling operation with a stride two (S-2). The second and third branches follow the same structure using 5 × 5 × 32 and 7 × 7 × 32 kernels, respectively, enabling the extraction of lesion characteristics at multiple receptive fields. The 7 × 7 kernels are specifically introduced to capture larger and diffuse lesion patterns that may not be effectively represented by smaller kernels, addressing lesion-scale variability in plant disease images. To control computational overhead, only 32 filters are used in this branch.

In parallel, an additional down-sampling path applies a 5 × 5 × 32 convolution with S-2 in Conv.BN L7 to preserve complementary spatial information. The outputs of all parallel paths undergo a concatenation operation, yielding 128 × 128 × 128 feature maps (FMs). To control the model complexity, the concatenated features are projected using a 1 × 1 × 64 Conv.BN layer, reducing the representation to 128 × 128 × 64. In the second stage, the stem processes the projected features with several convolutional paths using different down-sampling strategies. In Conv.BN L9, a 3 × 3 × 32 kernel with stride S-1 is applied, followed by a 2 × 2 max-pooling layer with stride S-2, while an alternate path consists of Conv.BN L10 with a 3 × 3 × 32 kernel with stride S-2. To minimize the loss of information, a Conv.BN L8 with a 7 × 7 kernel and stride S-4 is applied directly to the original input image. The outputs from these paths are combined using concatenation to restore the context and increase the stability of features at lower resolutions. Finally, the concatenated FMs are fed to Conv.BN L14 where it uses a 3 × 3 kernel with 96 filters, followed by a max-pooling layer with S-2, to further compress spatial dimensions.

In parallel, features from Conv.BN L9 are routed through an additional refinement branch consisting of Conv.BN L11 (3 × 3, S-2) and Conv.BN L12 (3 × 3, S-1). The output of L12 is subsequently passed through Conv.BN L13 with S-2, alongside a parallel max-pooling operation, and the resulting features are concatenated. In the final stage of the stem, multiple low-resolution feature streams are aggregated to form a rich representation. The merged features from the refinement branch are processed by Conv.BN L16 using 5 × 5 kernels with 64 filters to enhance discriminative capacity. In parallel, Conv.BN L15 applies a 3 × 3 convolution with 64 filters and S-2, while Conv.BN L17 extracts coarse contextual features using 5 × 5 kernels with S-4 from first stage features. The outputs of these branches, together with the max-pooled features from Conv.BN L14, are concatenated to form a comprehensive multi-scale feature tensor of size 32 × 32 × 256. Finally, a 1 × 1 convolutional batch normalization layer is applied to compress the concatenated features to 128 channels, yielding a 32 × 32 × 128 output.

After the HFA stem, the FMs are refined using a Squeeze-Excitation Attention (SEA) Block to adaptively recalibrate channel-wise responses. First, GAP is applied across spatial dimensions, transforming the input from 32 × 32 × 128 to a compact 1 × 1 × 128 descriptor that summarizes global contextual information for each channel. This descriptor is then passed through a Fully Connected (FC) layer that reduces the channel dimension to 1 × 1 × 16 using a reduction ratio r = 8, followed by a Leaky ReLU activation to introduce non-linearity and preserve gradient flow. The reduced representation is subsequently expanded through a second FC layer to restore the original channel dimensionality, resulting in a 1 × 1 × 128 attention vector. A sigmoid activation is applied to normalize the attention weights. Finally, these channel-wise weights are multiplied element-wise with the original 32 × 32 × 128 FMs, yielding recalibrated output features.

The output of the SEA Block is subsequently processed by Multi-scale Residual (MsR) Block-A to enhance feature representation across multiple receptive fields while preserving spatial resolution. This block comprises several parallel transformation paths operating on the same input. Each path begins with a 1 × 1 Conv.BN layer with 64 channels, which serves as a channel projection to reduce computational complexity and enable efficient multi-scale processing. The projected features are then processed using Conv.BN layers with different receptive fields, including 3 × 3 × 128 and 5 × 5 × 128 convolutions with dilation rate R = 1, as well as a 3 × 3 × 128 convolution with dilation rate R = 2, allowing the network to capture both fine-grained local patterns and broader contextual information associated with plant disease symptoms. In parallel, a 3 × 3 max-pooling layer with S-1 is applied to preserve spatial resolution while improving robustness to local variations. The outputs of all parallel paths are fused through element-wise addition, forming a residual aggregation that maintains the original feature dimensionality.

The output of MsR Block-A, with dimensions 32 × 32 × 128, is subsequently processed by (Reduction-Expansion) RE Block-A, which is designed to achieve spatial down-sampling while preserving discriminative information. In conventional CNN architectures, spatial reduction is typically performed using max-pooling layers alone, which can lead to irreversible information loss, particularly for fine-grained visual patterns such as small lesions and texture variations in plant disease images. To mitigate this limitation, the proposed RE Block replaces standalone max pooling with a structured multi-branch down-sampling strategy that jointly reduces spatial resolution and expands channel capacity. Specifically, the input FMs are processed through multiple parallel paths. Two convolutional branches first apply 1 × 1 Conv.BN layers with 32 channels to perform channel projection and reduce computational cost. These projected features are then passed through 3 × 3 × 64 and 5 × 5 × 64 Conv.BN layers, respectively, each with S-2. In parallel, a 2 × 2 max-pooling layer with S-2 provides an additional down-sampling pathway that preserves salient spatial responses. The outputs of all branches are subsequently concatenated. Through this combined RE strategy, the block transforms the input from 32 × 32 × 128 to 16 × 16 × 256, simultaneously reducing the spatial dimensions and doubling the number of feature channels.

The output of RE Block-A, with dimensions 16 × 16 × 256, is subsequently refined using a SEA Block that strengthens channel-wise feature recalibration. The attention-enhanced features are then passed to Multi-scale Residual Block-B, which follows the same multi-branch design as Block-A but processes a larger number of FMs, enabling richer multi-scale representation learning at deeper network levels while preserving the spatial resolution at 16 × 16. The output of MsR Block-B remains 16 × 16 × 256 and is subsequently processed by RE Block-B, which extends the RE strategy to deeper layers by reducing the spatial dimensions and doubling the channel depth, producing FMs of size 8 × 8 × 512. These high-capacity features are further refined by a SEA Block, which adaptively recalibrates the increased number of channels to emphasize disease-relevant information. GAP is then applied to aggregate spatial information into a compact 1 × 1 × 512 feature vector, reducing model complexity and enhancing generalization. To mitigate overfitting, a dropout rate of 0.2 is applied before the final classification stage. Finally, a Softmax layer outputs class probability distributions for plant disease prediction.

#### 3.4.1. The Activation Function

The activation function plays a crucial role in CNN training by introducing nonlinearity and transforming the input signal into the output signal. Traditional activation functions such as Sigmoid, Tanh, and ReLU have certain limitations that can impact the training process. Sigmoid and Tanh activation functions suffer from the issue of gradient vanishing. Additionally, Sigmoid involves complex power operations, which slow down the training. Although, ReLU activation function has a strong convergence rate but can lead to the problem of dead neurons as the number of layers increases [17].

To address these limitations, researchers proposed the state-of-the-art Swish [28], and Hard Swish [29] activation functions. The proposed HFA-Net architecture utilized the Hard Swish activation function throughout the convolutional layers.

The Hard Swish activation function is expressed by the following Equation (1):

(1) $$Hard\;Swish\;(x)=x\frac{ReLU6\left(x+3\right)}{6}$$

The Hard Swish activation function poses several benefits over other activation functions. Firstly, it provides a high convergence rate during training, enabling faster and more efficient learning. Secondly, it alleviates the gradient vanishing problem during backpropagation. The Hard Swish activation function in the HFA-Net architecture extracts more effective features and enhances the training process.

#### 3.4.2. The Loss Function

This research utilized the focal loss function to fine-tune the model using training images and their labels. This loss function addresses the category imbalance problem in PDD. It minimizes the loss by assigning different weights to samples based on their difficulty and the category imbalance. The focal loss function ensures that low sampled or challenging categories get more importance. By incorporating the focal loss function (Equation (2)), the proposed framework effectively handles category imbalance and improves the model’s ability to accurately classify plant diseases across various categories, including challenging and rare cases.

(2) $$L\left(Y_{act},\;f\left(x\right)\right)=-\frac{1}{N}\sum_{i=1}^{N}\sum_{j=1}^{C}\;\beta {\left(1-f_{j}\right)}^{\alpha }\;y_{ij}log(f_{j})$$

This formulation is a modification of the standard cross-entropy loss used in classification problems, particularly designed to address class imbalance, where $L\left(Y_{act},\;f\left(x\right)\right)$ represent the loss for a set of predictions $f\left(x\right)$ compared to the actual values $Y_{act}$. It introduces a modulating factor ${\left(1-f_{j}\right)}^{\alpha }$ to decrease the loss contribution from easy examples and tunable parameters set to α=2 and β=0.25 to control this adjustment.

### 3.5. Interpretability Analysis Using Grad-CAM

To enhance model transparency and support reliable decision-making, the proposed HFA-Net integrates Grad-CAM [15] as an XAI technique. Grad-CAM generates class-specific localization maps that identify spatial regions within an input image that contributes most significantly to the predicted class [16,17]. In the proposed architecture, Grad-CAM is applied to the final convolutional block preceding the GAP layer, where high-level semantic features are preserved while maintaining spatial structure. Given an input image and a target class c, the gradient of the class score $y^{c}$ with respect to the feature maps ($FM^{k}$) of the selected convolutional layer is computed. The channel-wise importance weight $\alpha_{k}^{c}$ for each feature map k is obtained by performing GAP over the spatial dimensions of the gradients as shown in Equation (3):

(3) $$\alpha_{k}^{c}=\frac{1}{H\times W}\sum_{i=1}^{H}\;\sum_{j=1}^{W}\;\frac{\partial \;y^{C}}{\partial FM_{i,j}^{k}}$$

where H and W denote the height and width of the FMs. These weights quantify the contribution of each channel to the target class prediction. The Grad-CAM heatmap is then computed as a weighted linear combination of the FMs followed by a Leaky ReLU activation as described in Equation (4):

(4) $$L_{Grad-CAM}^{c}=Leaky\_ReLU\sum_{i=1}^{H}\alpha_{k}^{c}\;FM^{k}\;$$

The resulting heatmap is upsampled to the input image resolution and overlaid on the original image, visually highlighting disease-affected regions that drive the model’s prediction. This interpretability analysis confirms that HFA-Net focuses primarily on lesion-specific structures rather than background artifacts, reinforcing its reliability under real-world agricultural conditions.

### 3.6. Experimental Setup and Evaluation Protocol

To evaluate the performance of the proposed HFA-Net, all experiments were conducted using an Intel(R) Core (TM) i7-7700K CPU with a 4.50 GHz Max Turbo Frequency and 32 GB of RAM. The model training was executed on the NVIDIA Tesla T4 GPU, which boasts 40 streaming multiprocessors, a 6 MB L2 cache, and 16 GB of GDDR6 memory, providing high computational speeds necessary for handling complex CNN operations. The training process spanned 60 epochs with a batch size of 32, employing the Adam optimizer for dynamic adjustment of learning rates. A systematic approach was adopted to save the best model weights based on their validation performance. These optimized weights were then used during the final evaluation and testing phase, ensuring the HFA-Net performed optimally under various conditions. Furthermore, to ensure a fair and unbiased comparison, all models were trained and evaluated under identical experimental conditions, including the same preprocessing pipeline, dataset splits, and training configurations.

### 3.7. Test-Time Augmentation (TTA)

Test-Time Augmentation (TTA) was used to test the models under realistic illumination variability. Unlike training-time augmentation, TTA was applied solely at inference to replicate realistic changes in lighting that are often experienced in the field. The proposed HFA-Net and the current baseline models were tested on the original test set and illumination-augmented test samples. The variation in illumination was simulated by direct element-wise pixel intensity scaling of the test images. In order to simulate high-illumination (bright) conditions, pixel values were boosted by 15% and 25%, and low-illumination (dark) conditions were simulated by decreasing pixel values by 15% and 25%. These regulated brightness variations maintain structural and textural information and add realistic global lighting variations, which allow a systematic evaluation of model stability in different illumination levels. This TTA strategy offers a uniform and equitable system of evaluation of robustness between models and enables the performance degradation or stability to be directly analyzed under extreme lighting conditions. The protocol to be used in the evaluation includes original, lightened, and darkened test samples, which makes the evaluation protocol closer to the real-life situation in agriculture imaging, where the illumination conditions are not always predictable and homogeneous.

### 3.8. Performance Evaluation Metrics

To assess the efficacy of the proposed framework and other CNN classifiers, standard metrics were used, including recall, precision, F1-score, and overall accuracy. These metrics are reported for each class label, their calculations are derived from the Confusion Matrix (CM), and the CM[i][j] is the number of instances in class i and predicted to be in class j.

- CM[i][i] represents the True Positive (TP) samples for class i.
- $\sum_{j=1,\;j\neq i}^{n}CM[j][i]\;$represents the False Positive (FP) samples for class i, which are instances predicted as class i but belong to other classes.
- $\sum_{j=1,\;j\neq i}^{n}CM[i][j]\;$represents the False Negative (FN) samples for class i, these instances are actually in class i, but are predicted as other classes.

With these definitions, the performance metrics for each class are described in Equations (5)–(8).

(5) $$Accuracy=\frac{\sum_{i=1}^{n}CM[i][i]\;}{\sum_{i=1}^{n}\sum_{j=1}^{n}CM[i][j]}\;$$

(6) $${Precision}_{i}=\frac{{TP}_{i}}{{TP}_{i}+{FP}_{i}}=\frac{CM[i][i]}{CM[i][i]+\sum_{j\neq i}CM[j][i]}$$

(7) $${Recall}_{i}=\frac{{TP}_{i}}{{TP}_{i}+{FN}_{i}}=\frac{CM[i][i]}{CM[i][i]+\sum_{j\neq i}CM[i][j]}$$

(8) $${F1-Score}_{i}=\;\frac{2\times {Precision}_{i}\times {Recall}_{i}}{{Precision}_{i}+{Recall}_{i}}$$

where n is the number of classes.

To evaluate the computational efficiency of the proposed HFA-Net and other CNN classifiers, several complexity metrics were considered, including the number of parameters, GFLOPs, and inference latency as described in Equations (9)–(13).

(9) $${\;Params}_{conv}=\;\left(K_{h}\times K_{w}\times C_{in}\times C_{out}\right)+biases$$

(10) $${Params}_{total}(M)=\frac{\sum_{l=1}^{L}{Params}_{l}}{10^{6}}$$

(11) $$FLOPs=\left({2\times H}_{out}\times W_{out}\times C_{out}\right)\times \left(K_{h}\times K_{w}\times C_{in}\right)$$

(12) $${GFLOPs}_{total}(M)=\frac{\sum_{l=1}^{L}{Params}_{l}}{10^{6}}$$

(13) $$Latency\;(ms)=\frac{{Time}_{total}}{N}\times 1000$$

where $K_{h}$ and $K_{w}$ are the height and width of the convolution kernel, respectively. $C_{in}$ and $C_{out}$ represent the number of input and output channels. $H_{out}$ and $W_{out}$ denote the spatial dimensions of the output feature map. $Time_{total}$ represents the total inference time measured in seconds, while N denotes the total number of test images used for latency computation.

Finally, a quantitative evaluation of HFA-Net explanation faithfulness was conducted using perturbation-based metrics, as defined in Equations (14)–(17). The deletion metric evaluates how rapidly the model confidence decreases when the most relevant pixels are progressively removed. At step k, the top k% most important pixels are replaced with a baseline value (i.e., blurred image), producing a perturbed image $x_{del}^{\left(k\right)}$. The corresponding confidence is

(14) $$D(k)=f_{c}\left(x_{del}^{\left(k\right)}\right)$$

The Deletion AUC is computed as

(15) $${AUC}_{del}=\frac{1}{K-1}\sum_{k=1}^{K-1}\frac{D(k)+D(k+1)}{2}$$

The insertion metric evaluates how rapidly the model confidence increases when the most relevant pixels are progressively added to a baseline image $x_{base}$ (i.e., blurred image). At step k, the top k% pixels are inserted, producing $x_{ins}^{\left(k\right)}$:

(16) $$I(k)=f_{c}\left(x_{ins}^{\left(k\right)}\right)$$

The Insertion AUC is computed as:

(17) $${AUC}_{ins}=\frac{1}{K-1}\sum_{k=1}^{K-1}\frac{I(k)+I(k+1)}{2}$$

## 4. Results and Comparative Analysis

### 4.1. Ablation Study of HFA-Net

Table 2 shows an ablation study that assesses the contributions of architectural components of the proposed HFA-Net framework.

The baseline configuration that uses SFF stem with MsRB, ReLU activation, and cross-entropy loss achieves an accuracy of 85.92%, indicating the limitations of conventional CNN design. The inclusion of the REB improves the classification performance to 87.83%. A major performance gain is observed when the SFF stem is replaced with the proposed HFA stem, increasing accuracy to 91.79%, which demonstrates the effectiveness of early multi-scale feature extraction. Further improvement is observed by replacing ReLU with Swish, increasing the accuracy to 92.25%. A comparable result is obtained with Hard-Swish 92.23%. This improvement highlights the advantage of smoother gradient propagation, while Hard-Swish additionally offers lower computational complexity during the forward pass. Introducing the SEAM achieves a significant improvement and increases the accuracy to 93.91%, confirming that channel-wise feature recalibration is important in highlighting the disease-relevant representations. Substituting cross-entropy loss with focal loss further enhances the performance to 94.21%. The addition of MACB helps to deal with dataset imbalance and leads to further improvement in accuracy to 94.87%, which shows the importance of balanced training data in model learning. Finally, the integration of IACE gives the highest accuracy of 96.03% and hence shows the critical role of mitigating illumination variance and improving generalization.

### 4.2. Robustness of HFA-Net Without IACE Under Illumination Variability

**Figure 8:**
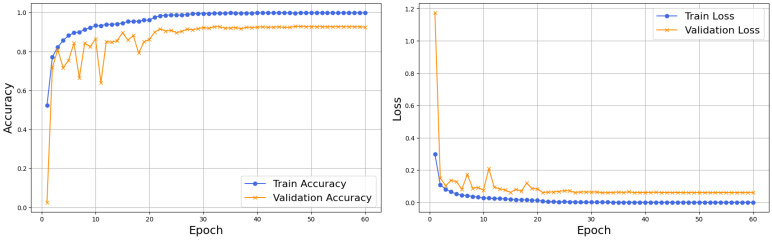
Training and validation accuracy and loss curves of HFA-Net without IACE.

Figure 8 shows the training and validation accuracy and loss curve of HFA-Net without IACE. The training accuracy elevates quickly in the first few epochs and slowly converges toward saturation, which shows the effective feature learning. Validation accuracy shows a similar pattern, with small variations in early epochs, and then it stabilizes on a high level, which reflects a good generalization. Similarly, the training loss drops sharply and converges to near-zero values, while the validation loss shows an early decrease and mild oscillations, then converges. The close match between training and validation curves suggests that HFA-Net has stable learning behavior with no severe overfitting.

**Figure 9:**
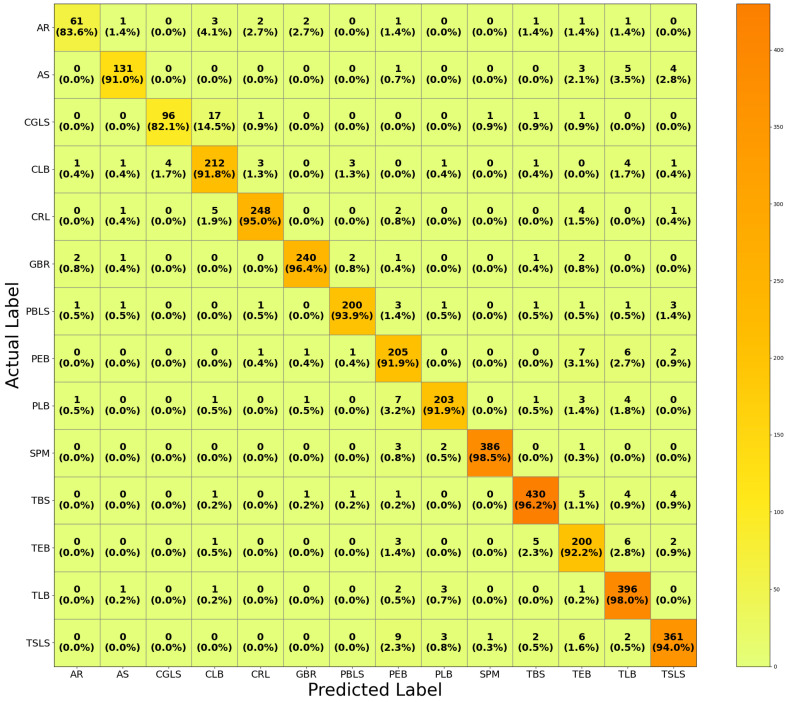
Confusion matrix of HFA-Net on the original test set without IACE.

Figure 9 shows the CM derived from the evaluation of HFA-Net on the original test set, without TTA and IACE preprocessing, that gives a detailed class-wise performance evaluation under nominal illumination conditions. The performance results show consistently high TP rates across most of the disease categories, which is indicative of the high discriminative capability of the model when trained and tested under standard lighting conditions. Classes like SPM, TBS, TLB, GBR, and CRL show particularly high correct classification rates, which are evidence of effective learning of disease-specific visual characteristics. Misclassifications are low and mainly occurred between visually similar classes of disease, such as CGLS–CLB and PEB–PLB, where overlapping lesion morphology and texture patterns of the diseases present inherent challenges.

Table 3 summarizes the class-wise performance of HFA-Net on the original test samples. The results show high scores for precision, recall, and F1-score for most of the classes, showing stable and well-balanced classification behavior under nominal lighting conditions. Notably, the model has a good balance between sensitivity and specificity for all the classes without severe performance degradation for any class.

Table 4 shows the HFA-Net’s average performance on the original test set with an average precision of 93.88%, a recall of 92.59%, and an F1-score of 93.15%, corresponding to the overall accuracy of 94.21%. These high values confirm the model’s effectiveness in capturing disease-related features, in the scenario when the training and testing illumination conditions are closely matched. Under reduced illumination, for TTA: Dark 15%, the model still has high performance of 92.82% precision, 92.20% recall, 92.38% F1-score and a 93.48% accuracy, which shows good low illumination resistance. However, by using TTA: Dark 25%, the model achieved a precision of 89.49%, a recall of 88.56%, an F1-score of 88.74%, and an accuracy of 89.68%. Under severe low-lighting conditions, the model still maintains acceptable performance level showing a certain robustness even without using any preprocessing. An asymmetrical trend is seen under increased illumination. For TTA: Bright 15%, HFA-Net achieves 92.40% precision, 91.12% recall, 91.72% F1-score and 92.95% accuracy, which means that the effect of brightness amplification is not too strong. In contrast, TTA: Bright 25% shows that precision decreases to 90.21%, recall decreases to 87.80%, F1-score decreases to 88.66%, and accuracy decreases to 90.05%. Across all the illumination scenarios, the overall average 91.76% precision, 90.47% recall, 90.93% F1-score, and 92.07% accuracy demonstrate HFA-Net stable behavior.

### 4.3. Robustness of HFA-Net with IACE Under Illumination Variability

**Figure 10:**
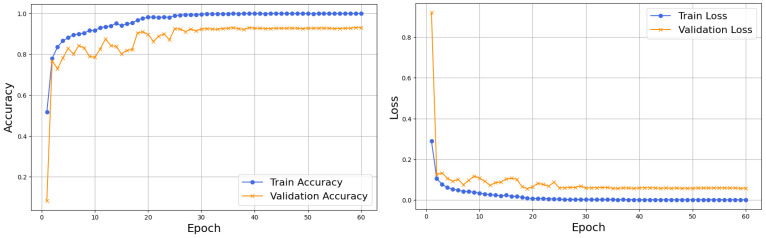
Training and validation accuracy and loss curves of HFA-Net with IACE preprocessing.

The accuracy and loss curves of HFA-Net using IACE preprocessing are shown in Figure 10. The model is characterized by a high convergence rate where training accuracy and validation accuracy follow a steady trend with a minor fluctuation as compared to the non-IACE setup. During the initial epochs, both the training and the validation losses decrease quickly, indicating the stable optimization. The minor difference between training and validation curves indicates that IACE is beneficial to reduce variability caused by illumination, which allows more stable learning and increases resistance to different lighting conditions.

**Figure 11:**
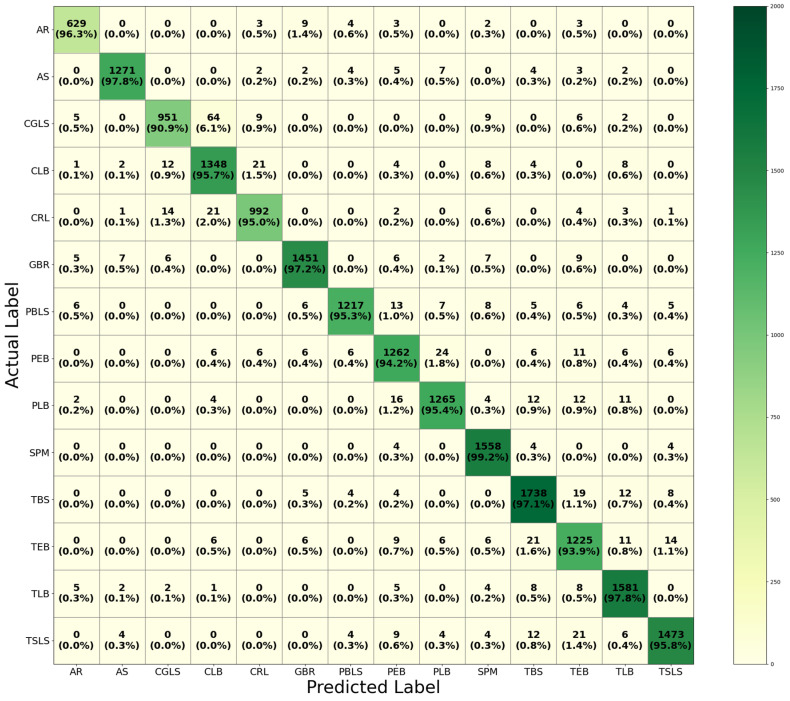
Confusion matrix of HFA-Net evaluated on the original test set after IACE.

The CM in Figure 11 shows the results of HFA-Net after IACE preprocessing and gives a class-wise assessment. The results demonstrate consistent high TP rates in all categories of diseases, which demonstrates that IACE is efficient at standardizing illumination without modifying disease-specific visual cues. Classes, which were moderately confused in non-IACE conditions, including CGLS–CLB, PEB–PLB and TEB–TBS, show less misclassification, reflecting better understanding of visually similar symptoms. Diseases with characteristic patterns such as SPM, GBR, CRL, and TLB attain good recognition performance, which indicates the consistency of discriminative features after IACE preprocessing. In general, this CM provides more reliable performance as compared to the non-IACE case and proves that illumination-adaptive preprocessing improves the class-wise reliability even in the standard test conditions.

Table 5 describes the performance of HFA-Net, evaluated on the original test samples after IACE preprocessing, and highlights the impact of illumination-adaptive normalization. The model demonstrates high precision, recall, and F1-score across all disease categories, which proves that IACE is a reliable pipeline. Classes that previously showed sensitivity to visual similarity and contrast variations, such as CGLS, CLB, and TEB, demonstrate notable improvements in recall and balanced precision after preprocessing. Overall, these findings indicate that IACE significantly enhances the reliability of classes in the nominal test conditions, which supports the idea of further testing that can be conducted in the case of simulated illumination variability.

Table 6 shows that HFA-Net achieves its highest overall performance on the original test samples after applying IACE, with an average precision of 96.06%, recall of 95.85%, and F1-score of 95.94%, corresponding to an accuracy of 96.03%. These findings show that illumination-adaptive preprocessing is effective in normalizing contrast and luminance differences, which result in the production of highly balanced sensitivity and specificity by class. In the case of TTA: Dark 15%, the HFA-Net achieved 93.00% precision, 92.33% recall, 92.55% F1-score, and 93.68% accuracy. That means that moderate darkening has been effectively compensated by IACE. The model has stable behavior even at TTA: Dark 25% where its precision, recall and F1-score are above 91%, and accuracy is 92.95%. These findings underscore the fact that IACE significantly reduces the negative impact of low-light conditions by maintaining lesion contrast and structural features. The same tendency is noted under high illumination simulations. In the case of TTA: Bright 15%, HFA-Net attained 94.06% precision, 93.37% recall, 93.62% F1-score, and 94.63% accuracy, which reflects its stability under moderate brightness amplification. Under more extreme conditions (TTA: Bright 25%), performance shows a controlled decline, with precision at 92.56%, recall at 91.45%, and F1-score at 91.91%, while accuracy remains above 93%. Across all illumination scenarios, the overall averages of 93.59% precision, 92.96% recall, 93.19% F1-score, and 94.07% accuracy demonstrate that the integration of IACE significantly improves illumination robustness and generalization across diverse lighting environments.

### 4.4. HFA-Net Explainability Analysis

The explainability analysis provides both quantitative and qualitative evidence that the proposed HFA-Net focuses on disease-relevant lesion regions rather than irrelevant background patterns.

**Figure 12:**
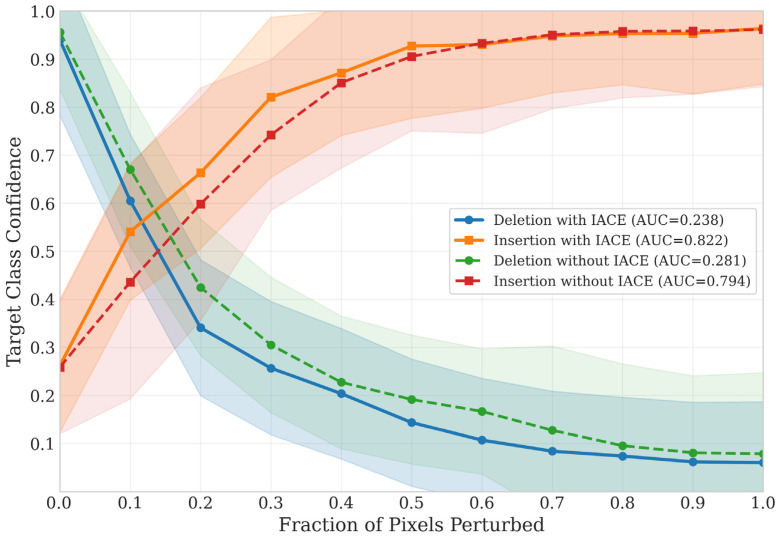
XAI-based deletion and insertion curves for HFA-Net with and without IACE.

As illustrated by the perturbation curves in Figure 12, both deletion and insertion behaviors confirm the reliability of the learned explanations. In the deletion setting, the confidence curve of HFA-Net without IACE already shows a clear and progressive decline as salient pixels are removed, indicating that the highlighted regions are genuinely important for the model’s predictions. This demonstrates that the proposed architecture itself learns meaningful pathological representations. Moreover, the integration of IACE further enhances the trustworthiness of these explanations. With IACE, the deletion curve drops more steeply, while the insertion curve rises more rapidly and attains higher confidence levels at earlier perturbation stages, indicating that the explanation maps become more concentrated on truly discriminative lesion structures after illumination normalization.

These trends are quantitatively supported by Table 7, where the Deletion AUC decreases from 0.281 to 0.238, and the Insertion AUC increases from 0.794 to 0.822 after incorporating IACE. A similar pattern is observed in the mean confidence values: the mean deletion confidence decreases from 0.302 to 0.262, whereas the mean insertion confidence increases from 0.778 to 0.823. Collectively, these findings indicate that HFA-Net delivers faithful visual explanations, while IACE further improves their precision, consistency, and interpretability under variable illumination conditions.

**Figure 13:**
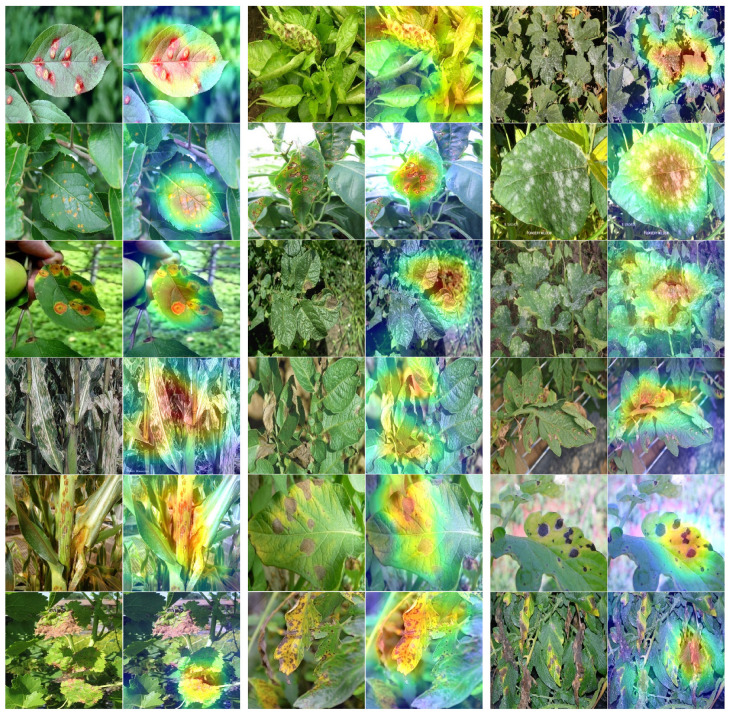
Grad-CAM visualizations of HFA-Net’s predictions on original test samples without IACE.

The qualitative Grad-CAM visualizations further reinforce these quantitative observations. Figure 13 illustrates Grad-CAM heatmaps generated from HFA-Net predictions without illumination-adaptive preprocessing. The visualizations reveal that the network is mostly focusing on regions of the lesion that are relevant to the disease and not by on background artifacts.

**Figure 14:**
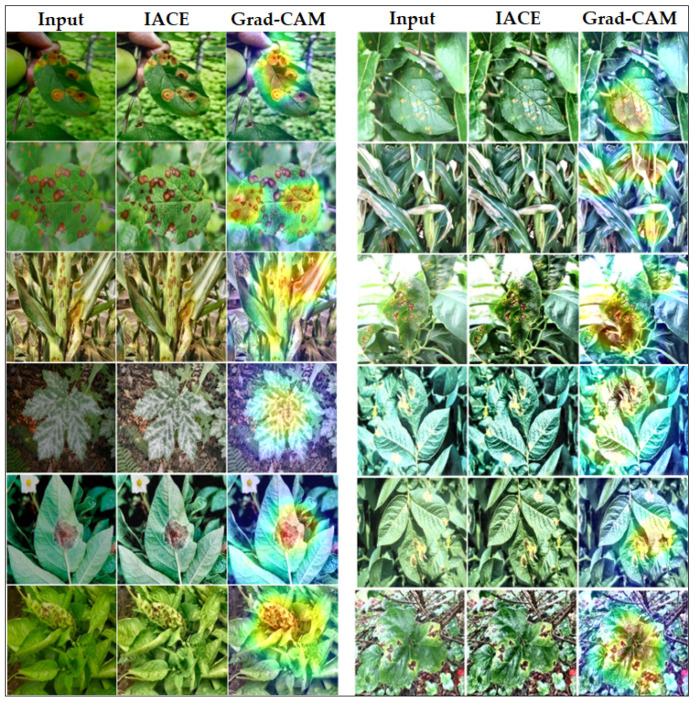
Qualitative explainability analysis of HFA-Net with IACE under illumination variability.

Figure 14 demonstrates the effect of IACE on stabilizing visual appearance under both bright and dark TTA conditions and its impact on model interpretability. After IACE preprocessing, contrast and luminance inconsistencies are effectively normalized, leading to clearer lesion boundaries and enhanced disease-relevant structures. The Grad-CAM maps of the corresponding images highlight pathological areas on the leaves while ignoring the background and illumination artifacts.

### 4.5. Comparative Analysis Without IACE Under Illumination Variability

Table 8 presents the comparative accuracy of HFA-Net and state-of-the-art architectures without applying IACE preprocessing. Under nominal illumination, all models benefit from matched training and testing distributions. MobileNet-V2 (81.32%) demonstrates the least performance, which indicates the weaknesses of depthwise separable convolutions and the lower representational power of the model in fine-grained disease texture recognition. VGG-16 (85.63%) and AlexNet (89.37%) have better accuracy due to the use of more convolutional layers but are limited by the inability to fuse the features across scales. ResNet-18 (90.91%) and ResNet-50 (91.44%) are more accurate than VGG-16, which proves the benefit of residual learning, though further depth does not bring significant gains. Parallel multi-scale convolutions are beneficial for Inception-V3 (92.67%), and it is better than residual models.

DenseNet-121 (93.12%) and DenseNet-201 (93.31%) again optimize through dense feature reuse, which allows smoother gradient flow and representation sharing. Xception-Net (91.83%) is also a competitive one, but the variant of DenseNet is ahead of it because it has the scarcity of explicit feature aggregation. HFA-Net has been shown to be the most accurate one with a score of 94.21%, surpassing all the baselines through the strategic combination of heterogeneous multi-scale aggregation, residual processing, and controlled feature expansion.

In the presence of a moderate decrease in illumination (TTA: Dark 15%), MobileNet-V2 (77.85%) is significantly affected, which suggests that this model is very sensitive to contrast reduction. Feed-Forward CNNs, VGG-16 (84.26%) and AlexNet (88.78%) decay less compared to MobileNet-V2. ResNet-18 (87.64%) and ResNet-50 (87.22%) have similar performance, indicating that residual connections stabilize the learning process, but they do not entirely compensate for illumination loss. The multi-branch design, used in Inception-V3 (89.82%) is beneficial, and it performs better than the residual networks. DenseNet-121 (90.52%) and DenseNet-201 (90.57%) are very robust, as they have dense connectivity and reuse of features. Xception-Net (90.01%) works similarly to DenseNet-121. Compared to other state-of-the-art CNNs, HFA-Net performed much better, achieving 93.48%, with the least relative degradation and better retention of discriminative information in moderate low-light conditions.

Under the 15% bright TTA condition, MobileNet-V2 (76.88%) proves that it is susceptible to high illumination. VGG-16 (84.17%) and AlexNet (88.45%) are stable but not as effective as deeper and multi-scale architectures. ResNet-18 (88.70%) is a little higher than ResNet-50 (87.41%), indicating that deeper residual networks have the possibility of enhancing noise during brightness increase. Inception-V3 (89.71%) has been steady, and DenseNet-121 (90.35%), DenseNet-201 (90.69%), and Xception-Net (90.41%) are the most solid baseline architectures. The highest accuracy is achieved by HFA-Net (92.95%), which shows that its heterogeneous feature aggregation and RE strategy and efficient to preserve salient features despite partial overexposure.

Immense darkening (i.e., 25% dark TTA) is a major challenge to any CNN architecture. The proposed HFA-Net achieves the highest classification accuracy of 89.68%, outperforming all baseline architectures under low-illumination field conditions. Lightweight models such as MobileNet-V2 (72.74%) exhibit significant performance degradation, indicating sensitivity to brightness reduction, while deeper residual networks, including ResNet-50 (78.80%) and ResNet-18 (80.62%), fail to maintain robust feature discrimination under reduced contrast. Although DenseNet variants (82.75% for DenseNet-121 and 82.33% for DenseNet-201) and VGG-16 (82.63%) demonstrate moderate stability due to dense feature propagation, AlexNet achieves 87.75% accuracy, likely benefiting from larger early convolutional kernels that capture broader texture patterns. However, its performance is still inferior to HFA-Net.

In extreme brightness scenario (TTA: Bright 25%), fine textures and lesion boundaries are suppressed by overexposure. The largest degradation is seen in MobileNet-V2 (72.07%). The sensitivity of VGG-16 (81.99%) and ResNet-50 (78.61) is significant, whereas ResNet-18 (83.86%) is more stable, which indicates that moderate depth can be beneficial in saturation. DenseNet-121 (83.92%), DenseNet-201 (85.26%), and Xception-Net (85.54%) are the best models exploiting reused and multi-path features. However, HFA-Net attains 90.05%, which is the most accurate performance achieved and the least relative performance decline at this extreme illumination. This finding validates that the architectural design of HFA-Net offers a better resistance to extreme variations in brightness.

The overall accuracy, which averages performance across the original, moderate, and extreme illumination conditions, provides a consolidated measure of model robustness under realistic variability. MobileNet-V2 records the lowest overall accuracy (76.17%), confirming that lightweight architectures struggle under illumination perturbations. VGG-16 (83.74%) improves stability through deeper convolutional stacks but remains limited by its single-scale design. ResNet-50 (84.70%) and ResNet-18 (86.35%) demonstrate that residual learning enhances generalization, with the shallower ResNet-18 showing greater robustness than its deeper counterpart. Inception-V3 (87.26%) benefits from multi-branch feature extraction, outperforming residual networks, while DenseNet-121 (88.13%), DenseNet-201 (88.43%), AlexNet (88.35%), and Xception-Net (88.45%) form the strongest baseline group due to multi-scale features, dense connectivity, and effective feature reuse. In contrast, HFA-Net achieves the highest overall accuracy of 92.07%, surpassing all competing models by a significant margin, which confirms its superior robustness and consistent performance across all illumination scenarios without relying on illumination-adaptive preprocessing.

**Figure 15:**
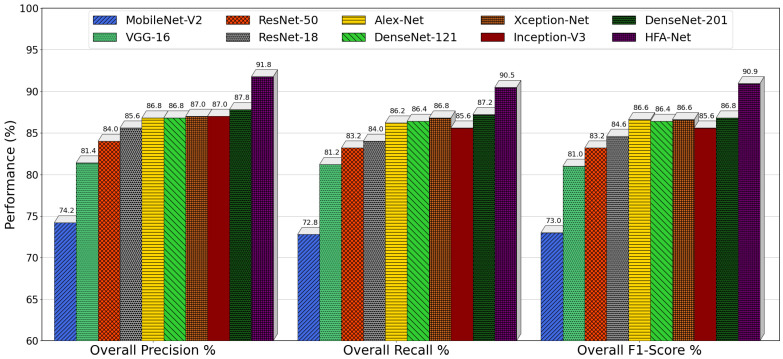
Overall performance comparison with state-of-the-art CNN models without IACE.

Figure 15 presents a combined comparison of overall precision, recall, F1-score, and accuracy across all models, summarizing performance averaged over the original test set and all illumination-augmented scenarios. These findings clearly separate lightweight, conventional, and advanced architectures based on their classification behavior. MobileNet-V2 has the lowest scores in each of the measures, having 74.20% precision, 72.80% recall, 73.00% F1-score, and 76.17% accuracy, indicating its poor representational ability and lower robustness to changes in illumination. VGG-16 accomplishes the performance of 81.40% precision, 81.20% recall, and 83.74% accuracy, but the single-scale nature limits additional improvements. The residual architectures record a stable improvement, with ResNet-50 recording 84.00% precision and 84.70% accuracy, while ResNet-18 records a slight improvement in robustness (85.60% precision, 86.35% accuracy) and thus it is observed that moderate depth is more robust when it comes to lighting condition generalization. Multi-scale and densely connected models demonstrated improved performance. Inception-V3 achieves 87.00% and 87.26% precision and accuracy, respectively, which is advantaged by parallel convolutional paths, whereas DenseNet-121, DenseNet-201, AlexNet, and Xception-Net constitute a solid cluster with an 86–88% range of precision and recall, with accuracy close to 88.5%. On the other hand, HFA-Net is always the most effective among all competing models, with the precision of 91.76%, recall of 90.47%, F1-score of 90.93%, and accuracy of 92.07%. The significant margin in all four metrics is confirmation that HFA-Net not only enhances accuracy but also possesses a better balance between precision and recall that exhibits better generalization and strong performance under diverse illumination conditions than state-of-the-art CNN architectures.

### 4.6. Comparative Analysis with IACE Under Illumination Variability

After applying IACE preprocessing, all models demonstrate improved performance compared to their non-IACE counterparts, confirming the effectiveness of illumination normalization, as shown in Table 9. MobileNet-V2 increases substantially to 88.81%, though it remains constrained by limited representational capacity. VGG-16 (88.03%) and AlexNet (88.92%) benefit moderately from IACE but continue to lag behind deeper and multi-scale architectures. Residual models achieve stronger results, with ResNet-50 (90.04%) slightly outperforming ResNet-18 (89.82%), indicating that moderate depth generalizes better under illumination normalization. More advanced architectures show further gains, with Xception-Net (91.24%), Inception-V3 (92.64%), DenseNet-201 (93.23%), and DenseNet-121 (93.40%) forming the top-performing baseline group. HFA-Net achieves the highest accuracy at 96.03%, demonstrating that when combined with illumination-adaptive preprocessing, its architectural advantages are further amplified.

Under moderate darkening (TTA: Dark 15%), all models exhibit enhanced robustness relative to the non-IACE setting. MobileNet-V2 (81.82%) shows the largest relative improvement but remains the weakest performer. VGG-16 (87.78%), AlexNet (87.53%), and ResNet-18 (88.23%) maintain stable performance, while ResNet-50 (87.41%) shows slightly lower robustness. Multi-scale and densely connected architectures, which include Xception-Net (90.46%), Inception-V3 (90.99%), DenseNet-201 (92.56%), and DenseNet-121 (92.31%), perform notably better, indicating strong resilience to moderate illumination loss. HFA-Net achieves 93.68% accuracy, outperforming all baselines and exhibiting minimal degradation, confirming that the integration of IACE with heterogeneous feature aggregation effectively preserves lesion visibility under low-light conditions.

Under severe darkening (TTA: Dark 25%), performance decreases across models, yet the relative ranking remains consistent. MobileNet-V2 drops sharply to 70.00%, while VGG-16 (86.35%), ResNet-18 (85.77%), and AlexNet (85.65%) retain moderate robustness. Dense and multi-branch models again outperform residual architectures, with DenseNet-121 (90.01%) and DenseNet-201 (89.62%) showing strong stability. HFA-Net achieves the highest accuracy at 92.95%, demonstrating superior resilience under extreme low illumination even after illumination normalization.

Under moderate brightness amplification (TTA: Bright 15%), most models maintain stable performance, reflecting the effectiveness of IACE in mitigating overexposure effects. MobileNet-V2 (85.46%) improves significantly but remains less competitive. VGG-16 (86.83%), AlexNet (87.83%), ResNet-18 (89.12%), and ResNet-50 (87.50%) show moderate robustness. Advanced architectures again lead, with Xception-Net (90.77%), Inception-V3 (91.27%), DenseNet-201 (92.42%), and DenseNet-121 (92.73%). HFA-Net records 94.63% accuracy, maintaining a clear margin over all baselines.

Under severe brightness increase (TTA: Bright 25%), performance degradation becomes more pronounced, particularly for lightweight and residual models, such as MobileNet-V2 (79.56%) and ResNet-50 (81.26%). DenseNet variants and Xception-Net remain more stable, achieving accuracy close to 90%, while HFA-Net performs 93.07%, showing the smallest relative drop. The overall accuracy, averaged across all illumination conditions, further confirms this trend, with HFA-Net achieving at 94.07%, which demonstrates the combined effectiveness of IACE and the proposed architectural design for illumination-robust plant disease classification.

The overall accuracy, which aggregates performance across the original test set as well as all illumination-augmented scenarios, provides a comprehensive measure of robustness after IACE preprocessing. MobileNet-V2 has the lowest total accuracy (81.13%), which means that illumination normalization cannot completely offset its low feature extraction ability. VGG-16 (86.91%) and AlexNet (87.20) exhibit moderate gains and highlight the importance of IACE. ResNet-18 (87.90%) performs better than ResNet-50 (85.70%), once again indicating that moderate depth generalizes better under illumination variability. More refined architectures such as Xception-Net (89.75%), Inception-V3 (90.27%), DenseNet-201 (91.50%), and DenseNet-121 (91.66%) illustrate the benefits of the processing pipeline. HFA-Net, in its turn, has the highest overall accuracy of 94.07, which is significantly higher than all state-of-the-art baselines. The outcome of this finding confirms that the combination of IACE preprocessing with heterogeneous feature aggregation, MsR learning, and RE design yields superior and consistent performance across diverse illumination conditions.

**Figure 16:**
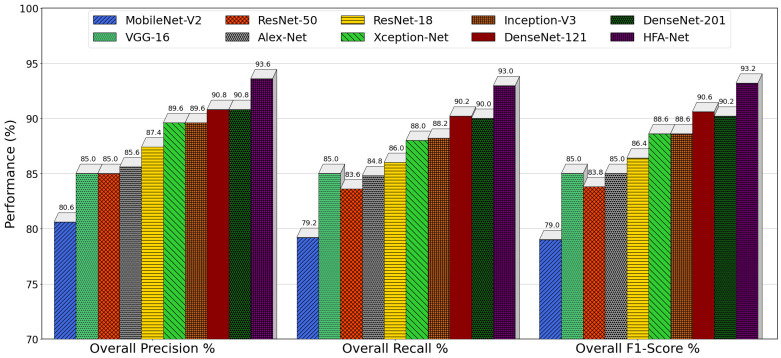
Overall performance comparison with state-of-the-art CNN models with IACE.

Figure 16 provides a consolidated comparison of overall precision, recall, and F1-score for all models after IACE preprocessing, summarizing performance averaged across original and illumination-augmented test sets. MobileNet-V2 has the worst performance in all measures, having 80.60% precision, 79.20% recall, and a 79.00% F1-score that underscores the weakness of lightweight architectures in illumination variability despite post-processing. ResNet-50 improves these metrics to 85.00% precision, 83.60% recall, and 83.80% F1-score, while VGG-16 achieves balanced precision, recall, and F1-score at 85.00%, indicating stable but limited generalization due to its SFF design. AlexNet and ResNet-18 achieve comparable performance, with ResNet-18 demonstrating slightly better balance (87.40% precision, 86.00% recall, and 86.40% F1-score), confirming the benefit of residual learning with moderate depth. Multi-scale architecture Xception-Net achieved 89.60% precision, 88.00% recall, and 88.60% F1-score; and Inception-V3 achieved 89.60% precision, 88.20% recall, and a 88.60% F1-score. DenseNet-201 and DenseNet-121 form the strongest baseline group, both exceeding 90% in precision, recall, and F1-score, reflecting the effectiveness of dense connectivity and feature reuse. In contrast, HFA-Net consistently outperforms all competing models, achieving 93.59% precision, 92.96% recall, and a 93.19% F1-score. The clear margin across all four metrics demonstrates not only superior accuracy but also a more favorable balance between sensitivity and specificity than existing CNN architectures under diverse illumination conditions.

### 4.7. Computational Complexity and Efficiency Analysis

The computational complexity of HFA-Net was compared with baseline CNN architectures in terms of parameter count, inference latency, and floating-point operations (GFLOPs). As shown in Table 10, classical deep networks such as VGG16 and AlexNet contain a large number of parameters, reaching 134.32 M and 57.34 M, respectively, which significantly increases memory consumption. In contrast, HFA-Net maintains a compact architecture with only 2.88 M parameters, representing a substantial reduction compared with most baseline models, including ResNet50 (23.62 M), Inception V3 (21.83 M), and DenseNet201 (18.36 M). Despite this lightweight design, the proposed model achieves the highest classification accuracy of 94.07%, demonstrating that the heterogeneous multi-scale feature aggregation and attention mechanisms effectively enhance feature representation without requiring a large number of parameters.

From a computational cost perspective, HFA-Net requires 16.41 GFLOPs and achieves an inference latency of 7.52 ms. Although the GFLOPs are higher than extremely lightweight architectures such as MobileNetV2 and ResNet18, the proposed network achieves a more favorable tradeoff between efficiency and predictive performance. Lightweight models typically exhibit lower computational cost but suffer from reduced classification performance, as reflected by the lower accuracy of MobileNetV2 (81.13%). Conversely, larger architectures such as DenseNet121 and DenseNet201 provide competitive accuracy but incur significantly higher latency. These results indicate that HFA-Net achieves an effective balance between model compactness, computational efficiency, and classification performance, making it well-suited for real-world precision agriculture systems where both accuracy and deployment efficiency are critical.

## 5. Conclusions

This work introduced HFA-Net, an illumination-adaptive and explainable framework for robust PDD, addressing core deployment barriers in field conditions, including lesion scale variability, inter- and intra-class similarity, class imbalance, and illumination fluctuations. HFA-Net architecturally differs from traditional feed-forward stems through a multi-level, multi-scale HFA stem, which allows it to capture early fine-to-coarse lesion cues. To improve feature preservation during down-sampling, the proposed Reduction–Expansion (RE) blocks expand channel depth while using 1 × 1 convolutional compression and Batch Normalization to maintain parameter efficiency and mitigate potential overfitting. For further regularization and stable convergence, Hard-Swish activation and Global Average Pooling (GAP) layers are incorporated. HFA-Net maintains high diagnostic accuracy under both high and low illumination conditions, producing illumination-invariant feature representations that improve robustness in field-deployed systems. To address illumination and contrast variations in agricultural environments, we design an illumination-adaptive preprocessing pipeline that integrates CLAHE and AGC. This standardization ensures consistent brightness and contrast across diverse lighting conditions. Moreover, a mean augmentation-based class balancing technique together with an enhanced focal loss function is employed to alleviate dataset imbalance. Grad-CAM is integrated to visualize the discriminative regions influencing model decisions, ensuring model transparency and interpretability. Illumination variability resistance was systematically measured with TTA that simulates realistic brightness shifts by scaling pixel intensities by ±15% and ±25%. Without preprocessing, HFA-Net maintained strong stability across lighting conditions, achieving 92.07% overall accuracy with balanced precision and recall, outperforming all competing CNN baselines under both dark and bright perturbations. When combined with the proposed IACE pipeline, performance improved further, reaching 94.07% overall accuracy and the highest overall precision (93.59%), recall (92.96%), and F1-score (93.19%) among all evaluated models, confirming that illumination normalization strengthens feature consistency and reduces degradation under extreme lighting. The ablation study substantiated the complementary role of each component, with the complete configuration achieving 96.03% accuracy on original test samples. Beyond quantitative gains, the integration of Grad-CAM provides transparent visual evidence that predictions are driven by lesion regions rather than spurious background cues, enhancing reliability for real-world decision support.

Despite its strong performance, the proposed framework has several limitations that should be acknowledged. First, the framework relies on image-based inputs that are sensitive to extreme noise or poor image quality, both of which are common in real-world agricultural environments. Second, although the combined dataset improves diversity by incorporating controlled and field-acquired samples, a degree of domain bias may persist and affect generalization to unseen environments. Third, the proposed illumination-adaptive preprocessing strategy primarily addresses intensity variations and does not explicitly model more complex lighting effects such as shadows or color temperature shifts.

Future research will extend HFA-Net by exploring hybrid architectures that integrate convolutional feature extractors with vision transformers to better capture long-range spatial dependencies and global contextual relationships. Investigating CNN-Transformer fusion strategies may further enhance robustness and generalization across heterogeneous agricultural datasets [36]. Additional validation on large-scale, field-acquired datasets from diverse geographic regions will be conducted to assess domain adaptability. Future evaluation protocols will also incorporate more realistic illumination variations, including color temperature shifts and non-uniform shadow effects, to better simulate complex real-world lighting conditions. Moreover, edge-aware optimization techniques will be explored to facilitate efficient deployment on resource-constrained agricultural devices, enabling real-time, field-level precision agriculture applications.

## Figures and Tables

**Table 1:** PlantVillageDoc benchmark dataset descriptions.

Image Label	Image Frequency	
	Pre Class-Balancing	Post Class-Balancing
Apple Rust (AR)	363	3267
Apple Scab (AS)	722	6498
Corn Gray Leaf Spot (CGLS)	581	5229
Corn Leaf Blight (CLB)	1173	7038
Corn Rust Leaf (CRL)	1305	5220
Grape Black Rot (GBR)	1244	7464
Pepper Bell Leaf Spot (PBLS)	1064	6384
Potato Early Blight (PEB)	1116	6696
Potato Late Blight (PLB)	1105	6630
Squash Powdery Mildew (SPM)	1962	7848
Tomato Bacterial Spot (TBS)	2237	8948
Tomato Early Blight (TEB)	1087	6522
Tomato Late Blight (TLB)	2020	8080
Tomato Septoria Leaf Spot (TSLS)	1921	7684

**Table 2:** Impact of different components on proposed framework performance.

Stem	MsRB	REB	SEAM	MACB	IACE	Activation Function	Loss Function	Accuracy %
SFF	✔	✘	✘	✘	✘	ReLU	Cross-entropy	85.92
SFF	✔	✔	✘	✘	✘	ReLU	Cross-entropy	87.83
HFA	✔	✔	✘	✘	✘	ReLU	Cross-entropy	91.79
HFA	✔	✔	✘	✘	✘	Swish	Cross-entropy	92.25
HFA	✔	✔	✘	✘	✘	Hard-Swish	Cross-entropy	92.23
HFA	✔	✔	✔	✘	✘	Hard-Swish	Cross-entropy	93.91
HFA	✔	✔	✔	✘	✘	Hard-Swish	Focal loss	94.21
HFA	✔	✔	✔	✔	✘	Hard-Swish	Focal loss	94.87
HFA	✔	✔	✔	✔	✔	Hard-Swish	Focal loss	96.03

✘: Component not included, ✔: Component included, IACE: Illumination-Adaptive Contrast Enhancement, MACB: Mean-Augmentation Class Balancing, SFF: Simple Feed-Forward, HFA: Heterogeneous Feature Aggregation, MsRB: Multi-scale Residual Block, REB: Reduction-Expansion Blocks, SEAM: Squeeze and Excitation Attention Mechanism.

**Table 3:** Class-wise performance of HFA-Net on the original test set without illumination augmentation.

Class	TP	FP	FN	TN	Pre.	Rec.	F1-Score
Apple Rust (AR)	61	5	12	3515	92.42	83.56	87.77
Apple Scab (AS)	131	6	13	3445	95.62	90.97	93.24
Corn Gray Leaf Spot (CGLS)	96	4	21	3480	96.00	82.05	88.48
Corn Leaf Blight (CLB)	212	29	19	3364	87.97	91.77	89.83
Corn Rust Leaf (CRL)	248	8	13	3328	96.88	95.02	95.94
Grape Black Rot (GBR)	240	5	9	3336	97.96	96.39	97.17
Pepper Bell Leaf Spot (PBLS)	200	7	13	3376	96.62	93.90	95.24
Potato Early Blight (PEB)	205	33	18	3371	86.13	91.93	88.94
Potato Late Blight (PLB)	203	10	18	3373	95.31	91.86	93.55
Squash Powdery Mildew (SPM)	386	2	6	3190	99.48	98.47	98.97
Tomato Bacterial Spot (TBS)	430	13	17	3146	97.07	96.20	96.63
Tomato Early Blight (TEB)	200	35	17	3376	85.11	92.17	88.50
Tomato Late Blight (TLB)	396	33	8	3180	92.31	98.02	95.08
Tomato Septoria Leaf Spot (TSLS)	361	17	23	3215	95.50	94.01	94.75

**Table 4:** Average Performance of HFA-Net on the original and illumination-augmented test sets without IACE.

	TTA: Dark 25%	TTA: Dark 15%	Original	TTA: Bright 15%	TTA: Bright 25%	Overall
Average Precision %	89.49	92.82	93.88	92.40	90.21	91.76
Average Recall %	88.56	92.20	92.59	91.12	87.80	90.47
Average F1-Score %	88.74	92.38	93.15	91.72	88.66	90.93
Accuracy	89.68	93.48	94.21	92.95	90.05	92.07

**Table 5:** Class-wise performance of HFA-Net on the original test set after IACE.

Class	TP	FP	FN	TN	Pre.	Rec.	F1-Score
Apple Rust (AR)	629	24	24	18,074	96.32	96.32	96.32
Apple Scab (AS)	1271	16	29	17,432	98.76	97.77	98.26
Corn Gray Leaf Spot (CGLS)	951	34	95	17,752	96.55	90.92	93.65
Corn Leaf Blight (CLB)	1348	102	60	17,355	92.97	95.74	94.33
Corn Rust Leaf (CRL)	992	41	52	17,711	96.03	95.02	95.52
Grape Black Rot (GBR)	1451	34	42	17,252	97.71	97.19	97.45
Pepper Bell Leaf Spot (PBLS)	1217	22	60	17,486	98.22	95.30	96.74
Potato Early Blight (PEB)	1262	80	77	17,441	94.04	94.25	94.14
Potato Late Blight (PLB)	1265	50	61	17,438	96.20	95.40	95.80
Squash Powdery Mildew (SPM)	1558	58	12	17,145	96.41	99.24	97.80
Tomato Bacterial Spot (TBS)	1738	76	52	16,965	95.81	97.09	96.45
Tomato Early Blight (TEB)	1225	102	79	17,478	92.31	93.94	93.12
Tomato Late Blight (TLB)	1581	65	35	17,122	96.05	97.83	96.93
Tomato Septoria Leaf Spot (TSLS)	1473	38	64	17,230	97.49	95.84	96.65

**Table 6:** Average performance of HFA-Net under illumination-normalized conditions after IACE preprocessing.

	TTA: Dark 25%	TTA: Dark 15%	Original	TTA: Bright 15%	TTA: Bright 25%	Overall
Average Precision %	92.29	93.00	96.06	94.06	92.56	93.59
Average Recall %	91.82	92.33	95.85	93.37	91.45	92.96
Average F1-Score %	91.91	92.55	95.94	93.62	91.91	93.19
Accuracy	92.95	93.68	96.03	94.63	93.07	94.07

**Table 7:** Quantitative evaluation of HFA-Net using perturbation-based faithfulness metrics.

Setting	Deletion AUC	Insertion AUC	Mean Deletion Confidence	Mean Insertion Confidence
Without IACE	0.281	0.794	0.302	0.778
With IACE	0.238	0.822	0.262	0.823

**Table 8:** Comparative accuracy of HFA-Net and state-of-the-art CNN models without IACE under varying illumination conditions.

	TTA: Dark 25%	TTA: Dark 15%	Original	TTA: Bright 15%	TTA: Bright 25%	Overall
MobileNet-V2 [30]	72.74	77.85	81.32	76.88	72.07	76.17
VGG-16 [31]	82.63	84.26	85.63	84.17	81.99	83.74
ResNet-50 [32]	78.80	87.22	91.44	87.41	78.61	84.70
ResNet-18 [32]	80.62	87.64	90.91	88.70	83.86	86.35
Inception-V3 [33]	80.23	89.82	92.67	89.71	83.89	87.26
DenseNet-121 [12]	82.75	90.52	93.12	90.35	83.92	88.13
Alex-Net [34]	87.75	88.78	89.37	88.45	87.39	88.35
DenseNet-201 [12]	82.33	90.57	93.31	90.69	85.26	88.43
Xception-Net [35]	84.45	90.01	91.83	90.41	85.54	88.45
HFA-Net	89.68	93.48	94.21	92.95	90.05	92.07

**Table 9:** Comparative analysis of accuracy under illumination variability with IACE preprocessing.

	TTA: Dark 25%	TTA: Dark 15%	Original	TTA: Bright 15%	TTA: Bright 25%	Overall
MobileNet-V2 [30]	70.00	81.82	88.81	85.46	79.56	81.13
ResNet-50 [32]	82.30	87.41	90.04	87.50	81.26	85.70
VGG-16 [31]	86.35	87.78	88.03	86.83	85.54	86.91
Alex-Net [34]	85.65	87.53	88.92	87.83	86.07	87.20
ResNet-18 [32]	85.77	88.23	89.82	89.12	86.55	87.90
Xception-Net [35]	88.31	90.46	91.24	90.77	87.95	89.75
Inception-V3 [33]	88.34	90.99	92.64	91.27	88.11	90.27
DenseNet-201 [12]	89.62	92.56	93.23	92.42	89.68	91.50
DenseNet-121 [12]	90.01	92.31	93.40	92.73	89.85	91.66
HFA-Net	92.95	93.68	96.03	94.63	93.07	94.07

**Table 10:** Computational complexity comparison of HFA-Net with baseline CNN architectures.

Model	Parameters (M)	Latency (ms)	GFLOPs	Overall Accuracy
VGG16	134.32	9.41	30.95	86.91
AlexNet	57.34	0.21	2.26	87.20
ResNet50	23.62	4.66	7.75	85.70
Inception V3	21.83	6.08	11.47	90.27
Xception Net	20.89	10.89	16.77	89.75
DenseNet201	18.36	30.17	8.63	91.50
ResNet18	11.20	1.63	3.66	87.90
DenseNet121	7.06	18.44	5.70	91.66
HFA-Net	2.88	7.52	16.41	94.07
MobileNetV2	2.28	1.64	0.61	81.13

## Data Availability

The datasets used in this study are publicly available and have been cited at the relevant locations within the manuscript. Access to the curated and processed versions of the data supporting the reported results can be obtained from the corresponding author upon reasonable request.
